# Local steroid activation is a critical mediator of the anti-inflammatory actions of therapeutic glucocorticoids

**DOI:** 10.1136/annrheumdis-2020-218493

**Published:** 2020-11-08

**Authors:** Chloe Fenton, Claire Martin, Rachel Jones, Adam Croft, Joana Campos, Amy J Naylor, Angela E Taylor, Myriam Chimen, Mark Cooper, Gareth G Lavery, Karim Raza, Rowan S Hardy

**Affiliations:** 1 Institute for Metabolism and Systems Research, University of Birmingham, Birmingham, UK; 2 Research into Inflammatory Arthritis Centre, Versus Arthritis, Institute of Inflammation and Ageing, University of Birmingham, Birmingham, UK; 3 MRC Arthritis Research UK Centre for Musculoskeletal Ageing Research, University of Birmingham Edgbaston Campus, Birmingham, UK; 4 Centre for Translational Inflammation Research, University of Birmingham, Birmingham, UK; 5 Centre for Endocrinology, Diabetes and Metabolism, University of Birmingham, Birmingham, UK; 6 Institute for Clinical Sciences, University of Birmingham, Birmingham, UK; 7 ANZAC Research Institute, The University of Sydney, Sydney, New South Wales, Australia; 8 Rheumatology, Sandwell and West Birmingham Hospitals NHS Trust, Birmingham, UK

**Keywords:** arthritis, arthritis, experimental, glucocorticoids, inflammation, synovitis

## Abstract

**Objectives:**

The enzyme 11β-hydroxysteroid dehydrogenase type 1 (11β-HSD1) plays a well-characterised role in the metabolism and activation of endogenous glucocorticoids (GCs). However, despite its potent upregulation at sites of inflammation, its role in peripheral metabolism and action of therapeutic GCs remains poorly understood. We investigated the contribution of 11β-HSD1 to the anti-inflammatory properties of the active GC corticosterone, administered at therapeutic doses in murine models of polyarthritis.

**Methods:**

Using the tumour necrosis factor-tg and K/BxN serum-induced models of polyarthritis, we examined the anti-inflammatory properties of oral administration of corticosterone in animals with global, myeloid and mesenchymal targeted transgenic deletion of 11β-HSD1. Disease activity and joint inflammation were scored daily. Joint destruction and measures of local and systemic inflammation were determined by histology, micro-CT, quantitative RT-PCR, fluorescence activated cell sorting and ELISA.

**Results:**

Global deletion of 11β-HSD1 resulted in a profound GC resistance in animals receiving corticosterone, characterised by persistent synovitis, joint destruction and inflammatory leucocyte infiltration. This was partially reproduced with myeloid, but not mesenchymal 11β-HSD1 deletion, where paracrine GC signalling between cell populations was shown to overcome targeted deletion of 11β-HSD1.

**Conclusions:**

We identify an entirely novel component of therapeutic GC action, whereby following their systemic metabolism, they require peripheral reactivation and amplification by 11β-HSD1 at sites of inflammation to deliver their anti-inflammatory therapeutic effects. This study provides a novel mechanistic understanding of the anti-inflammatory properties of therapeutic GCs and their targeting to sites of inflammation in polyarthritis.

**Key messages**

**What is already known about this subject?**

- Potent anti-inflammatory glucocorticoids such as prednisolone are rapidly metabolised, and circulate in both their active and inactive (prednisone) forms.
- Enzymes such as 11β-hydroxysteroid dehydrogenase type 1 (11β-HSD1), which is potently upregulated at sites of inflammation, reactivates inactive glucocorticoids such as prednisone.

**What does this study add?**

- This study demonstrates that following their oral delivery and systemic metabolism, the anti-inflammatory properties of active glucocorticoids are completely dependent on their peripheral reactivation by 11β-HSD type 1.
- The global deletion of 11β-HSD type 1 results in profound therapeutic glucocorticoid resistance.

**How might this impact on clinical practice or future developments?**

- This study provides a novel mechanistic understanding of the anti-inflammatory properties of therapeutic glucocorticoids and their targeting to sites of inflammation.

## Introduction

Due to their anti-inflammatory actions, therapeutic glucocorticoids (GCs) have been widely used in the management of inflammation. However, despite their continuing widespread use, several critical aspects of their therapeutic action remain unclear.^1^ The enzyme 11β-hydroxysteroid dehydrogenase type 1 (11β-HSD1) plays a well characterised role in the hepatic activation of structurally inactive GCs (such as cortisone and prednisone), converting them to their active counterparts (such as hydrocortisone and prednisolone).^2 3^ However, the role of 11β-HSD1 in mediating the anti-inflammatory, disease-modifying actions of therapeutic GCs remains poorly understood. This represents a significant barrier to our understanding of the mechanisms of action of therapeutic GC action in vivo and to the development of GCs with an enhanced benefit:risk ratio.

We explore the contribution of pre-receptor steroid metabolism by the enzyme 11β-HSD1 to the anti-inflammatory actions of GCs using in vivo models of chronic polyarthritis. We demonstrate a fundamental role for the peripheral re-activation of GCs in mediating their anti-inflammatory properties, with mice with global 11β-HSD1 deletion showing a complete resistance to their therapeutic effects of orally administered GCs in their active form. These findings change our understanding of how many structurally active therapeutic GCs elicit their anti-inflammatory effects, requiring peripheral reactivation by the enzyme 11β-HSD1, after their initial systemic inactivation, to mediate their beneficial immune-modulatory effects.

## Materials and methods

### Models of polyarthritis

The tumour necrosis factor (TNF)-tg model of chronic inflammatory polyarthritis, obtained courtesy of Professor George Kollias (BSRC Fleming, Athens), was maintained on a C57BL/6 background and compared with WT littermates.^4^ At day 32 of age, at the first onset of measurable polyarthritis, male TNF-tg mice received drinking water supplemented with either corticosterone (Cort) (100 µg/mL, 0.66% ethanol), or vehicle (0.66% ethanol) for 3 weeks. Mice were scored as previously described.^5 6^ At day 53, serum was collected by cardiac puncture and tissues excised for analysis. Serum transfer-induced arthritis (STIA) was induced by intravenous injection of 100 µL arthritogenic serum from KRN mice (K/BxN).^7^ Ankle or wrist joint thickness was monitored using callipers and reported as the change from baseline.

### Targeted deletion of 11β-HSD1

11β-HSD1 KO animals with global 11β-HSD1 deletion were crossed with TNF-tg animals to generate TNF‐tg^11βKO^ animals as previously described.^8^ Mesenchymal 11β-HSD1 KO animals were generated by crossing flx/flx-HSD11B1 mice with Twist2-cre mice to generate 11βHSD1flx/flx/Twist2cre animals, which were paired with TNF-tg animals to produce TNF-tg^11βHSD1flx/flx/Twist2cre^ (TNF-tg^11βflx/tw2cre^).^9–11^ Myeloid targeted 11β-HSD1 KO animals were generated by crossing flx/flx-HSD11B1 mice with LysM-cre mice to generate 11βHSD1flx/flx/LysMcre animals, which were paired with TNF-tg animals to produce TNF-tg^11βHSD1flx/flx/LysMcre^ (TNF-tg^11βflx/LysMcre^).^12^

### 11β-HSD1 activity

11β-HSD1 activity was determined by thin-layer chromatography as previously reported.^8 13^ Briefly, ex vivo tissue biopsies and in vitro cultures were incubated with 100 nmol/L of 11-dehydrocorticosterone (11-DHC) and tritiated [^3^H] tracer. Steroid conversion was measured using a Bioscan imager (Bioscan, Washington, District of Columbia, USA) and fractional conversion calculated.

### Primary fibroblast-like synoviocytes and macrophage culture

Primary fibroblast-like synoviocytes (FLS) were isolated from combined hind legs and front paws from mice following dissection and cleaning of tissue as previously reported.^13^ Briefly, joints were digested in RPMI containing 2% fetal calf serum (FCS), 2.5 mg/mL collagenase D (Roche) and 20 µg/mL DNase (Sigma‐Aldrich) for 45 min at 37°C with agitation. After filtering, cells were cultured in RPMI containing 10% FCS and 1% pen-strep and cultured to passage 3 before use. Primary murine peritoneal macrophages were isolated by CD11b+ve selection with CD11b MicroBeads (Miltenyi Biotec, Surrey, UK) following peritoneal lavage in phosphate-buffered saline, and maintained in Dulbecco's Modified Eagle Medium containing 10% FCS and 1% pen-strep and maintained for up to 48 hours.

### Gene expression analysis

Gene expression was assessed by TaqMan Gene Expression Assays (ThermoFisher Scientific) following mRNA isolation by innuPREP RNA Mini Kit (Analytikjena, Cambridge) and reverse transcription (Multiscribe, ThermoFisher Scientific) as per the manufacturer’s guidelines. Ccl2, cxcl2, Cxcl10, Tnfα, il1β, Il6 and gilz were determined using species-specific probe sets by real-time PCR on an ABI7500 system (Applied Biosystems, Warrington, UK). mRNA abundance was normalised to either 18S or Gapdh. Data, obtained as Ct values and ΔCt determined (Ct target–Ct 18S/GAPDH), were expressed as arbitrary units (AU) using the following transformation: (arbitrary units (AU)=1000×(2^−Δct^)).

### ELISA analysis

Serum interleukin (IL)-6 and corticosterone (R&D Systems, Abingdon, UK) were determined using commercially available ELISA assays in accordance with the manufacturer’s instructions.

### Histological analysis of joints

Histochemistry was performed on paraffin-embedded 10 µm sections. Pannus size at the humerus/ulna joint interface and osteoclast numbers on the bone surface pannus (following tartrate resistant acid phosphatase (TRAP) staining) were determined using ImageJ software as previously reported.^5 14^ For quantification, the mean of three adjacent 10 µm sections cut from the centre of the joint from six animals were assessed.

### MicroCT morphometry analysis

Front paws from mice were imaged using a Skyscan 1172 micro-CT scanner (Bruker) using X-ray beam settings of 60 kV/167 μA with a 0.5 mm aluminium filter. Projections were taken every 0.45° at 580 ms exposure. Image volumes were reconstructed using the Feldkamp algorithm (NRecon V.1.6.1.5, Bruker) having applied beam hardening correction. Front paws were reconstructed and MeshLab V.1.3.2 was used to generate meshes which could then be scored for bone erosions as described previously.^5^

### Serum steroid measurements

Serum samples were collected by cardiac bleeds to assess systemic metabolism between groups; 200 µL of serum was spiked with 0.2 ng of internal standard (corticosterone-d8 and cortisol-d4; purchased from Sigma-Aldrich, UK). Steroids were extracted via liquid-liquid extraction with 2 mL of tert-methyl butyl ether (MTBE). MTBE was evaporated to dryness under nitrogen at 55°C. Samples were reconstituted in 125 µL of 50/50 methanol/water for liquid chromatography tandem mass spectrometry analysis.^15 16^ Samples were measured on a Waters Xevo-XS mass spectrometer coupled to an Acquity uPLC with an electrospray ionisation source in positive ionisation mode. Steroids were identified by comparison to authentic reference standards, (Sigma-Aldrich), with matching retention time and identical mass transitions and quantified relative to a calibration series. Concentrations were calculated relative to internal standard corticosterone to corticosterone-d8 and 11-DHC and its isomer metabolite to cortisol-d4.

### Tissue digestion and flow cytometric analysis of synoviocytes

One hind leg and one front paw per mouse was dissected and cleaned of tissue as previously reported.^8^ Briefly, joints were digested in RPMI containing 2% FCS, 2.5 mg/mL collagenase D (Roche) and 20 µg/mL DNase (Sigma‐Aldrich) for 45 min at 37°C with agitation. After filtering, cells were centrifuged, red cells lysed and cells counted before being filtered through 40 µm cell strainer, incubated with anti-CD16/CD32 blocking antibody (1:200; eBioscience) for 10 min at RT, followed by staining with antibody cocktail at 4°C. Antibodies for membrane staining are outlined in online supplemental table 1). Data were acquired using a BD LSR Fortessa X20 and analysed using FlowJo software (FlowJo). The following gating strategy was used for myeloid cells: live cells were gated on CD45+CD11b+ cells. Neutrophils identified as Ly6g+, macrophages were Ly6g−, F4/80+ and inflammatory activated M1-like macrophages were F4/80+MHC class II+. T cells were identified as live CD45+CD3+. CD3+ cells were then stratified as CD4+ or CD8+ T cells. B cells were identified as CD45+CD3 and CD19+.

10.1136/annrheumdis-2020-218493.supp1 Supplementary data

### Statistical analysis

Statistical significance was defined as p<0.05 using either an unpaired Student’s t-test or two-way analysis of variance with Tukey post hoc analysis where a Gaussian distribution was identified.

## Results

### 11β-HSD1 KO animals are resistant to therapeutic GCs

**Figure 1:**
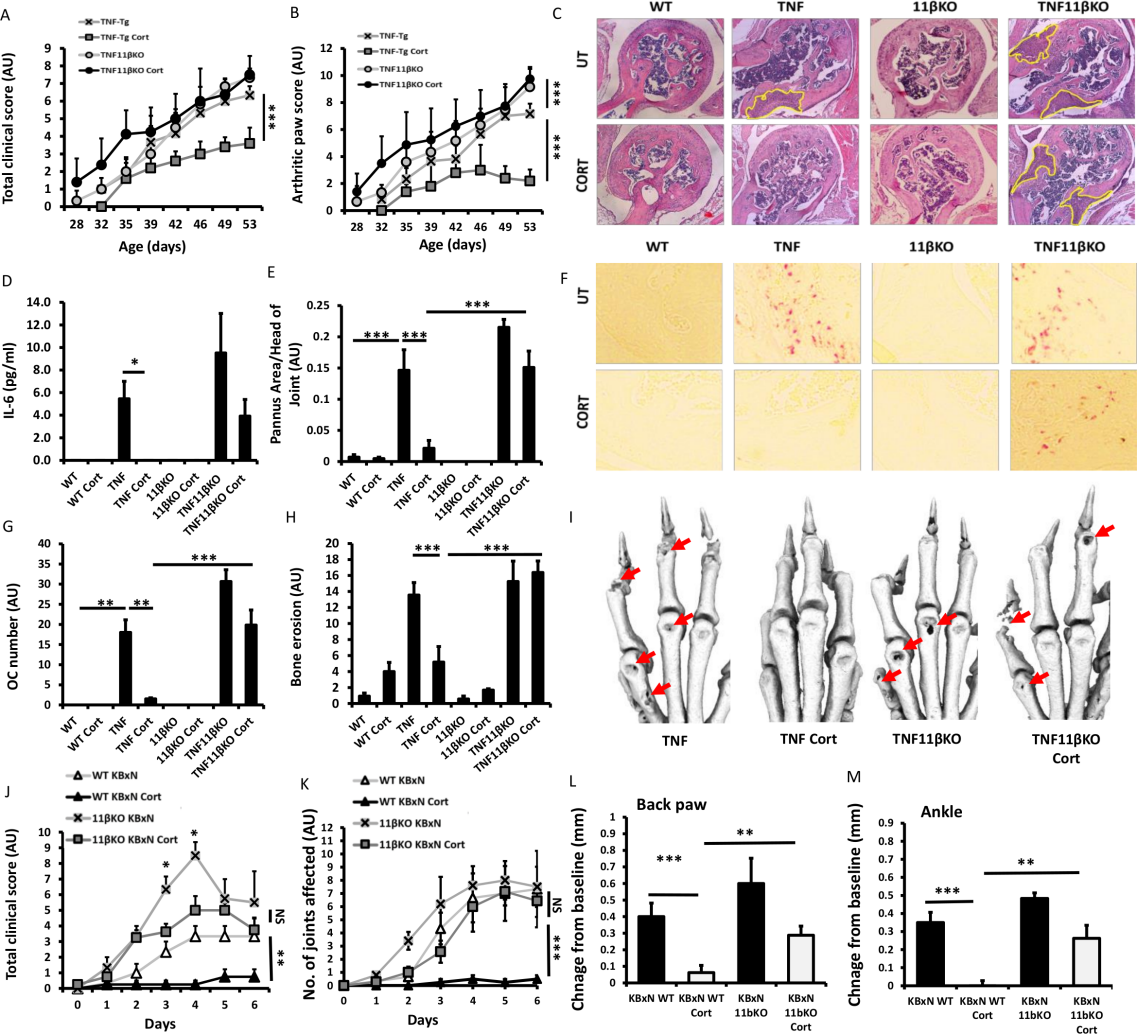
(A) Total clinical scores (arbitrary units (AU)), (B) arthritic paw scores (AU) and (C) representative images of synovitis at the ulna/humerus joint interface of wild-type (WT), tumour necrosis factor (TNF)-tg, 11βKO and TNF-tg^11βKO^ animals receiving either vehicle or corticosterone (100 µg/mL) in the drinking water for 3 weeks. (B) of TNF-tg and TNF-tg^11βKO^ animals receiving vehicle or corticosterone (100 µg/mL) in the drinking water for 3 weeks. (D) Serum levels of interleukin (IL)-6 determined by ELISA, (E) histological scoring of synovitis (AU), (F) representative images of TRAP stained osteoclasts at the ulna/humerus joint interface, (G) osteoclast number (AU) at the ulna/humerus joint interface, (H) quantification of bone erosion (AU) in the wrist, metacarpals and phalanges of WT, TNF-tg, 11βKO and TNF-tg^11βKO^ animals receiving either vehicle or corticosterone (100 µg/mL) in the drinking water for 3 weeks. (I) representative images of three-dimensional reconstructions of front paws of TNF-tg and TNF-tg^11βKO^ animals receiving vehicle or corticosterone (100 µg/mL) in the drinking water for 3 weeks, red arrows indicate erosions. (J) Total clinical scores (AU), (K) arthritic paw scores (AU) and swelling (mm) of (L) back paws and (M) ankles of WT and 11βKO animals after induction of arthritis with K/BxN serum receiving either vehicle or corticosterone (100 µg/mL) in the drinking water for 1 week. Values are expressed as mean±SE, n=6 per group for all TNF-tg experiments and n=5 (K/BxN), n=6 (K/BxN/Cort), n=6 (K/BxN^11βKO^) and n=5 (K/BxN^11βKO^/Cort). Statistical significance was determined using two-way analysis of variance with Tukey post hoc analysis. *P<0.05, **p<0.005, ***p<0.001.

We crossed the TNF-tg murine model of chronic polyarthritis onto the 11βKO background to generate TNF-tg animals with deletion of 11β-HSD1 (TNF‐tg^11βKO^). Wild-type (WT), TNF-tg, 11βKO and TNF‐tg^11βKO^ animals received either vehicle or corticosterone in drinking water at 50 and 100 µg/mL as previously reported.^2 14 17^ At 50 µg/mL, no significant change in disease activity or joint inflammation were apparent in TNF-tg animals and was discontinued from the study (online supplemental figure 1A‒C). At 100 µg/mL corticosterone resulted in a significant reduction in clinical scores and joint inflammation in TNF-tg animals (figure 1A, B). In contrast, anti-inflammatory effects of corticosterone were absent in TNF‐tg^11βKO^ animals. Similarly, serum IL-6 was reduced in TNF-tg mice receiving corticosterone (p<0.05), which was absent in TNF‐tg^11βKO^ counterparts (figure 1D). TNF-tg mice receiving corticosterone showed a marked reduction in pannus invasion and osteoclast numbers at the pannus bone interface, which was entirely absent in TNF‐tg^11βKO^ animals (figure 1C–G). Micro-CT analysis of juxta-articular erosions confirmed that corticosterone significantly reduced joint destruction in TNF-tg mice, but not TNF‐tg^11βKO^ animals (figure 1H, I). In the KBxN serum induction model of polyarthritis, similar patterns were observed with total clinical scores and joint inflammation scores being reduced by corticosterone in WT, but not in 11βKO animals at day 6 (figure 1J–M). Here, at early time points (days 3–4) GCs were able to partially suppress clinical scores of disease, including weight loss and lethargy in 11βKO animals, without impacting on measures of joint inflammation and swelling. These data demonstrate that GC activation by the enzyme 11β-HSD1 is a necessary step in mediating the anti-inflammatory actions of the GC corticosterone in the joints of animals with polyarthritis.

10.1136/annrheumdis-2020-218493.supp2 Supplementary data

### Oral corticosterone generates circulating 11-DHC substrate for 11β-HSD1 activation

**Figure 2:**
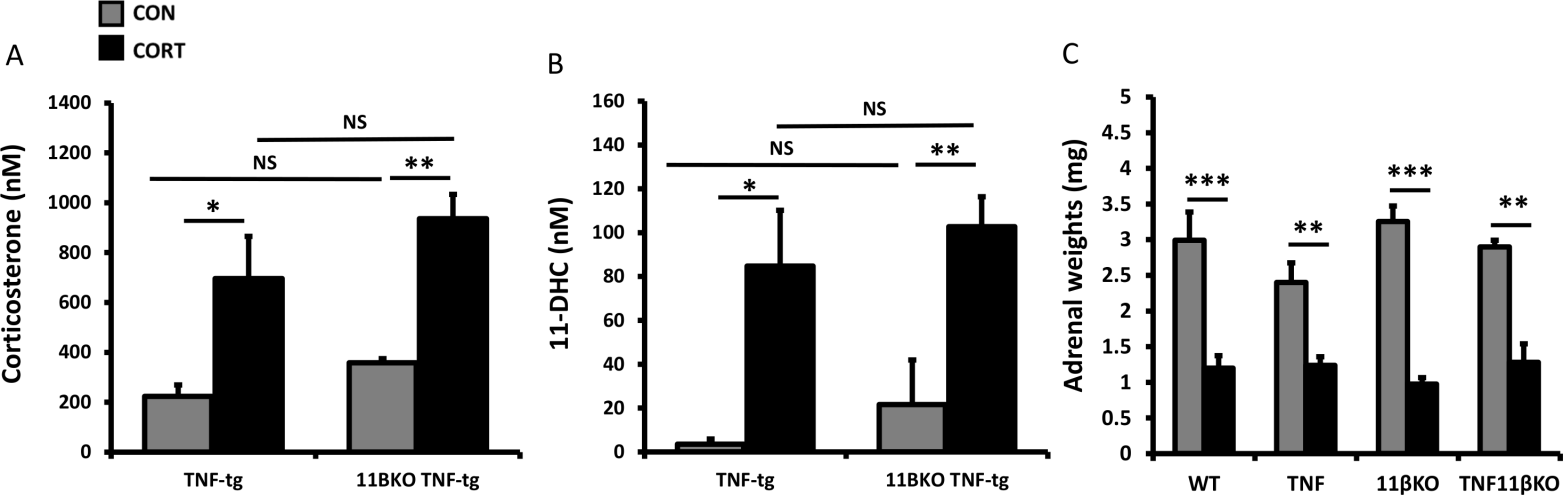
(A, B) Serum corticosterone and 11-dehydrocorticosterone (11-DHC) determined by liquid chromatography tandem mass spectrometry (LCMS) from tumour necrosis factor (TNF)-tg, 11βKO and TNF-tg11βKO animals receiving either vehicle or corticosterone (100 µg/mL) in the drinking water for 3 weeks. (C) Adrenal weights in wild-type (WT), TNF-tg, 11βKO and TNF-tg11βKO animals receiving either vehicle or corticosterone (100 µg/mL) in the drinking water for 3 weeks. Values are expressed as mean±SE, n=6 per group for ELISA and adrenal weights and n=3 per group for LCMS. Statistical significance was determined using two-way analysis of variance with Tukey post hoc analysis. *P<0.05, **p<0.005, ***p<0.001. NS, not significant.

Metabolism and inactivation of therapeutic GCs by renal 11β-HSD2 creates a circulating pool of inactive GC (corticosterone to 11-DHC in mice) available for peripheral activation by 11β-HSD1. To assess the systemic metabolism of oral administered corticosterone, we measured serum levels of the corticosterone and its inactive derivative 11-DHC. No differences were observed in daily intake of corticosterone between groups, determined by quantifying daily drinking water intake per mouse, with an average exposure of 22.5+1.44 µg/g of body weight. Here, serum corticosterone and 11-DHC were detected and significantly increased following administration of corticosterone, with exposure comparable across groups (figure 2A, B). In all groups, exposure to corticosterone resulted in a significant reduction in adrenal weights relative to vehicle (figure 2C). Analysis of corticosterone inactivation by the 11β-HSD enzymes within the synovium was assessed by thin layer chromatography in WT, TNF-tg, 11βKO, TNF-tg anNFtg^11βKO^ animals and showed no significant variation between groups (online supplemental figure 2). These data confirm a comparable increase in serum corticosterone and inactive 11-DHC in TNF-tg and TNF‐tg^11βKO^ animals receiving oral corticosterone.

10.1136/annrheumdis-2020-218493.supp3 Supplementary data

### Effects of oral corticosterone on leucocyte recruitment are dependent on 11β-HSD1

**Figure 3:**
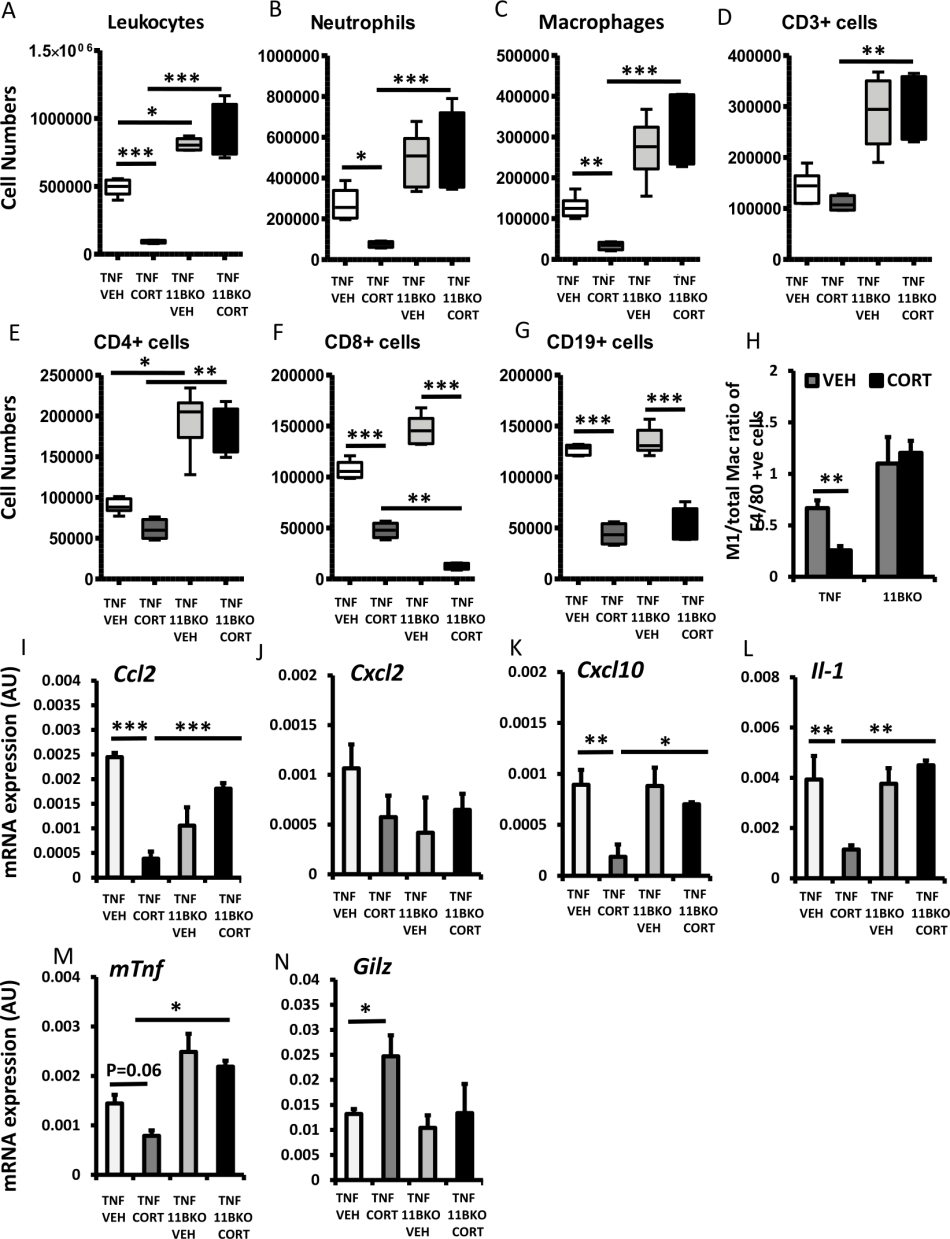
Cell numbers of (A) total leucocytes, (B) neutrophils, (C) macrophages, (D) CD3+ populations, (E) CD4+ populations, (F) CD8+ populations, (G) CD19+ populations and (H) the M1-like/total macrophage ratio determined by flow cytometry in tumour necrosis factor (TNF)-tg and TNF-tg^11βKO^ animals receiving vehicle or corticosterone (100 µg/mL) in the drinking water for 3 weeks. Gene expression (AU) of (I) Ccl2, (J) Cxcl2, (K) Cxcl10, (L) Il-1, (M) mTnf and (N) Gilz determined by quantitative PCR in tibia isolated from TNF-tg and TNF-tg^11βKO^ animals receiving vehicle or corticosterone (100 µg/mL) in the drinking water for 3 weeks. Values are expressed as mean±SE, n=6 per group. Statistical significance was determined using two-way analysis of variance with Tukey post hoc analysis. *P<0.05, **p<0.005, ***p<0.001.

We examined infiltrating leucocytes and inflammatory mediators in synovial tissue digests to assess their regulation by corticosterone. Here, while corticosterone resulted in a significant decrease in total leucocytes, neutrophils, macrophages, CD8+ and CD19+, but not CD3+ and CD4+ populations in TNF-tg mice, TNF‐tg^11βKO^ animals were resistant to the actions of corticosterone on many of these parameters, with no apparent reduction in total leucocytes, macrophages and neutrophils (figure 3A–D). TNF‐tg^11βKO^ animals receiving corticosterone also possessed significantly higher numbers of total leucocytes, neutrophils, macrophages, CD3+ (p<0.01) and CD4+ cell populations relative to TNF-tg counterparts receiving corticosterone (figure 3A–E). Here, corticosterone skewed macrophage polarisation, with reduced numbers of inflammatory activated M1-like polarised or macrophages relative to total macrophages, TNF-tg animals, while TNF‐tg^11βKO^ animals showed complete resistance to this effect (figure 3H). However, TNF‐tg^11βKO^ animals retained an effective suppression of both CD8+ and CD19+ cell populations in response to corticosterone (figure 3F–H). Analysis of gene expression in synovial tissue digests revealed a significant reduction in the chemokines Ccl2, *Cxcl10* and cytokine *Il-1b*, and an increased expression of anti-inflammatory *Gilz* expression in TNF-tg animals receiving corticosterone, which was entirely absent in TNF‐tg^11βKO^ animals (figure 3I–N). These data reveal that TNF‐tg^11βKO^ animals show marked resistance to the anti-inflammatory properties of therapeutic GCs on leucocyte recruitment and on regulation of local inflammatory mediators.

### Mice with stromal deletion of 11β-HSD1 retain anti-inflammatory responses to GCs

**Figure 4:**
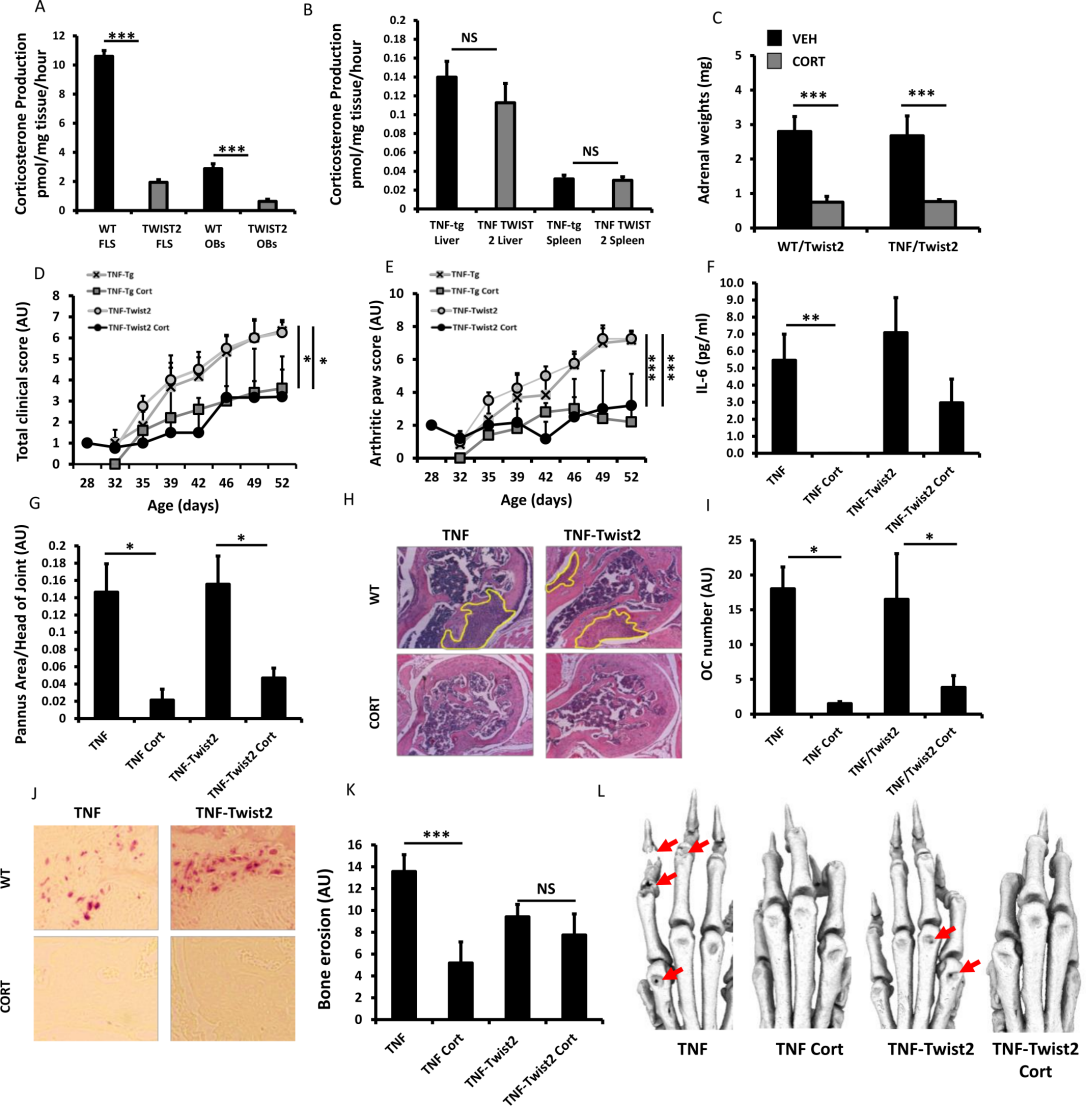
Corticosterone production (pmol/mg tissue/hour) in (A) fibroblast-like synoviocytes (FLS) and osteoblasts (OBs) cultures and (B) liver and spleen ex vivo biopsies isolated from tumour necrosis factor (TNF)-tg and TNF-tg^11βflx/tw2cre^ mice determined by scanning thin layer chromatography. (C) Adrenal weights (mg), (D) total clinical scores (AU), (E) arthritic paw scores (arbitrary units (AU)), (F) serum IL-6 determined by ELISA, (G) histological scoring (AU) and (H) representative images of synovitis at the ulna/humerus joint interface, (I) histological scoring (AU) and (J) representative images of TRAP stained osteoclast numbers at the ulna/humerus joint interface, (K) quantification of bone erosion (AU) in the wrist, metacarpals and phalanges and (L) representative images of three-dimensional reconstructions of front paws in TNF-tg and TNF-tg TNF-tg^11βflx/tw2cre^ animals receiving either vehicle or corticosterone (100 µg/mL) in the drinking water for 3 weeks. Values are expressed as mean±SE, n=6 per group. Statistical significance was determined using two-way analysis of variance with Tukey post hoc analysis. *P<0.05, **p<0.005, ***p<0.001.

Given the stromal upregulation of stromal 11β-HSD1 sites of inflammation, we wished to delineate its specific contribution to GC resistance in the TNF-tg^11βflx/tw2cre^ mouse relative to TNF-tg littermates, where we have previously reported effective mesenchymal deletion.^3 8^ A significant reduction in 11β-HSD1 activity was apparent in primary fibroblasts and osteoblasts isolated from TNF-tg^11βflx/tw2cre^ animals, while activity was retained in non mesenchyme derived tissues such as livers and spleen (figure 4A, B). Suppression of adrenal weights was apparent across all groups in response to corticosterone (figure 4C). Corticosterone significantly reduced clinical scores and measures of joint inflammation in both TNF-tg^11βflx/tw2cre^ and TNF-tg littermates (figure 4D, E). While circulating levels of the acute response cytokine IL-6, remained elevated in TNF-tg^11βflx/tw2cre^ receiving corticosterone, analysis of pannus invasion, osteoclast numbers and joint destruction by micro-CT, indicated that corticosterone was equally effective at suppressing disease activity in both TNF-tg and TNF-tg^11βflx/tw2cre^ animals (figure 4C, G–L). Consequently, despite effective deletion of 11β-HSD1 in the mesenchymal compartment, TNF-tg^11βflx/tw2cre^ animals retain a robust anti-inflammatory response to corticosterone.

### Partial GCs resistance with myeloid deletion of 11β-HSD1

**Figure 5:**
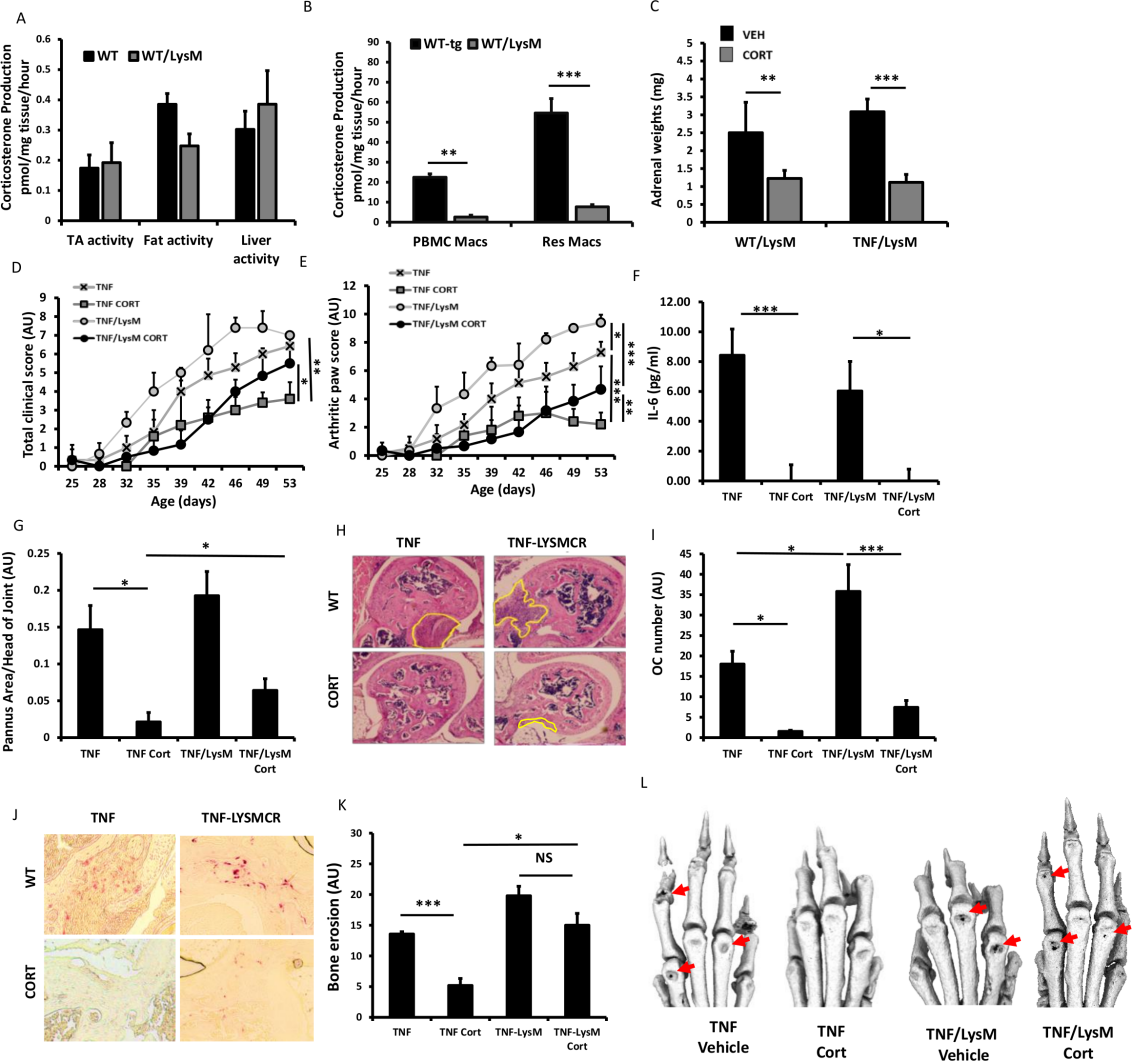
Corticosterone production (pmol/mg tissue/hour) in (A) tibialis anterior (TA) muscle, fat and liver ex vivo biopsies and (B) monocyte, peripheral blood mononuclear cell (PBMC)-derived macrophages and resident macrophages isolated from tumour necrosis factor (TNF)-tg and TNF-tg^11βflx/LysMcre^ mice determined by scanning thin layer chromatography. (C) Adrenal weights (mg), (D) total clinical scores (arbitrary units (AU)), (E) arthritic paw scores (AU), (F) serum interleukin (IL)-6 determined by ELISA, (G) histological scoring (AU) and (H) representative images of synovitis at the ulna/humerus joint interface, (I) histological scoring (AU) and (J) representative images of TRAP stained osteoclast numbers at the ulna/humerus joint interface, (K) quantification of bone erosion (AU) in the wrist, metacarpals and phalanges and (L) representative images of three-dimensional reconstructions of front paws in TNF-tg and TNF-tg^11βflx/LysMcre^ animals receiving either vehicle or corticosterone (100 µg/mL) in the drinking water for 3 weeks. Values are expressed as mean±SE, n=6 per group. Statistical significance was determined using two-way analysis of variance with Tukey post hoc analysis. *P<0.05, **p<0.005, ***p<0.001.

At sites of inflammation, 11β-HSD1 is highly expressed in macrophages and is implicated in regulating their anti-inflammatory properties.^3 18 19^ We used the LysMCre mouse (targeted towards neutrophils, macrophages and granulocytes) to generate tg^11βflx/LysMcre^ animals with a deletion of 11β-HSD1 in the myeloid compartment.^12^ 11β-HSD1 activity was significantly reduced in both peripheral blood mononuclear cell and peritoneal macrophages relative to WT counterparts (figure 5A). In contrast, normal 11β-HSD1 activity was apparent in tissues such as muscle, fat and liver (figure 5A, B). Corticosterone significantly reduced adrenal weights in both TNF-tg^11βflx/LysMcre^ animals and TNF-tg littermate controls of (figure 5C). TNF-tg^11βflx/LysMcre^ mice receiving corticosterone showed a significant reduction in joint inflammation scores but not in total clinical scores, while serum IL-6 levels were similarly decreased in both TNF-tg and TNF-tg^11βflx/LysMcre^ (figure 4D–F). A, significant reductions in both pannus size and osteoclast numbers were apparent in both TNF-tg and TNF-tg^11βflx/LysMcre^ animals receiving corticosterone (figure 5G–J). However, evidence of residual pannus and osteoclast numbers in TNF-tg^11βflx/LysMcre^ animals was supported by a greater incidence of juxta-articular joint destruction determined by micro-CT relative to TNF-tg counterparts (p<0.05) (figure 5K, L). These data demonstrate that mice with a myeloid targeted deletion of 11β-HSD1 retain the capacity to respond to therapeutic GCs.

### Paracrine GC signalling compensates for cell-specific 11β-HSD1 deletion

**Figure 6:**
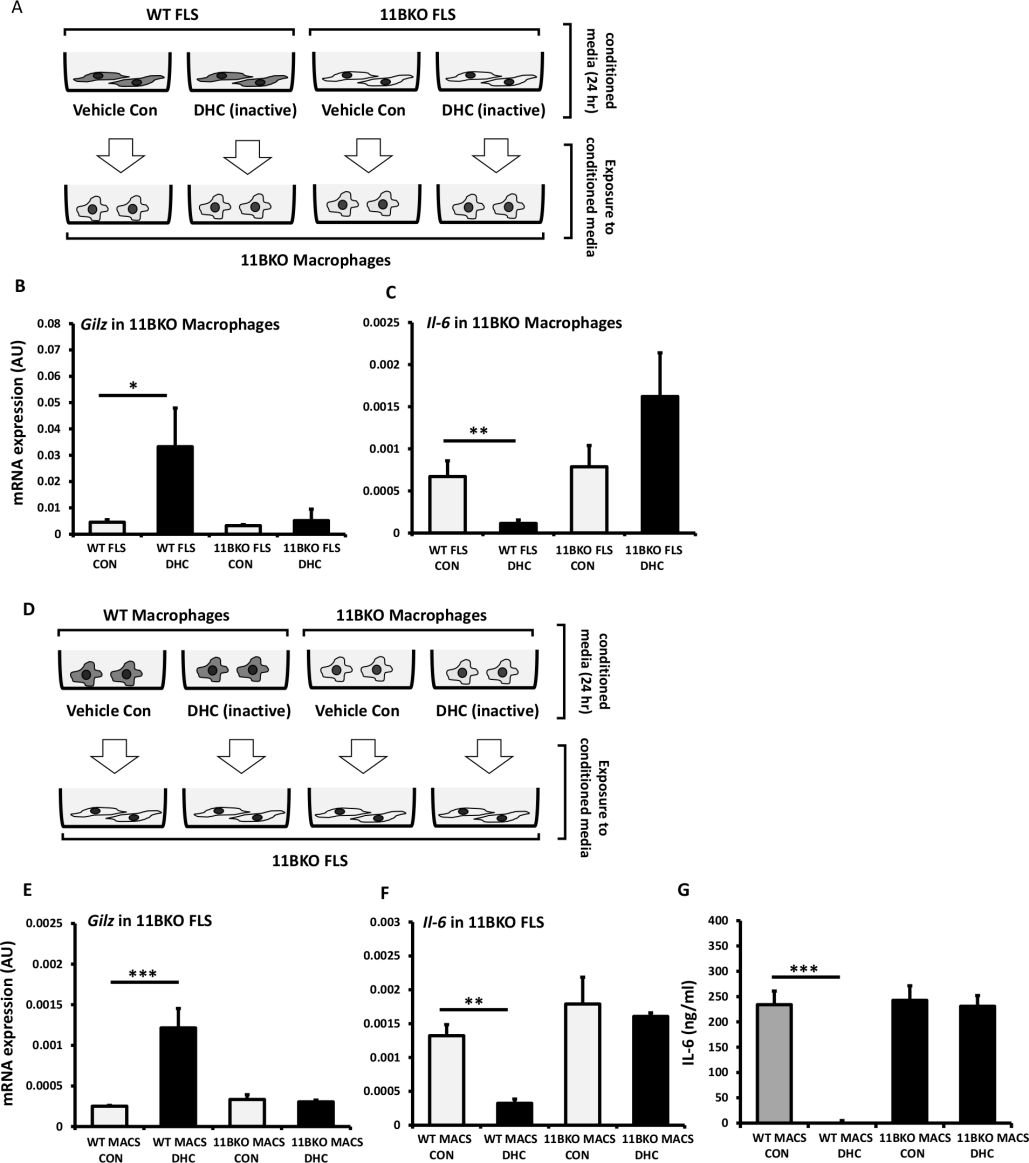
(A) Schematic representation of conditioned media experiments in which media from wild-type (WT) and 11βKO fibroblast-like synoviocytes (FLS) treated with either vehicle or dehydrocorticosterone (DHC) is used to treat 11βKO macrophages. Gene expression (arbitrary units (AU)) of (B) Gilz and (C) Il-6 in 11βKO macrophages treated with conditioned media from WT and 11βKO FLS determined by quantitative PCR (qPCR). (D) Schematic representation of conditioned media experiments in which media from WT and 11βKO macrophages treated with either vehicle or DHC is used to treat 11βKO FLS. Gene expression (AU) of (E) Gilz and (F) Il-6 in 11βKO FLS treated with conditioned media from WT and 11βKO macrophages determined by qPCR. (G) Protein levels of IL-6 (ng/mL) in the media of 11βKO FLS treated with conditioned media from WT and 11βKO macrophages determined by ELISA. Values are expressed as mean±SE, n=3 per group. Statistical significance was determined using one-way analysis of variance with Tukey post hoc analysis. *P<0.05, **p<0.005, ***p<0.001.

Given our findings in the stromal and myeloid targeted models, we performed co-culture experiments in FLS and macrophage to determine if GCs activated in one cell population could influence the other by paracrine signalling. We generated conditioned media by exposing WT and 11β-HSD1 KO FLS to the inactive GC 11-DHC for 24 hours, which was then placed on 11β-HSD1 KO macrophages for a further 24 hours prior to measuring GC response genes (figure 6A). Here, 11β-HSD1 KO macrophages responded to conditioned media from WT FLS exposed to 11-DHC (increasing Gilz and suppressing IL-6), but not conditioned media from 11β-HSD1 KO FLS (figure 6B, C). Conditioned media from WT and 11β-HSD1 KO macrophages conditioned with 11-DHC were then placed on 11β-HSD1 KO FLS for 24 hours and GC responsive gene analysed (figure 6D). 11β-HSD1 KO FLS responded to conditioned media from WT macrophages exposed to 11-DHC (increasing Gilz and suppressing IL-6), but did not respond to conditioned media generated in 11β-HSD1 KO macrophages (figure 6E, F). Similarly, IL-6 production in 11β-HSD1 KO FLS was suppressed in response to media from WT macrophages exposed to 11-DHC, but not from 11β-HSD1 KO macrophages exposed to 11-DHC (figure 6G). These data confirm that 11β-HSD1 can mediates paracrine GC signalling between distinct cell populations present at sight of inflammation, including macrophages and FLS.

## Discussion

**Figure 7:**
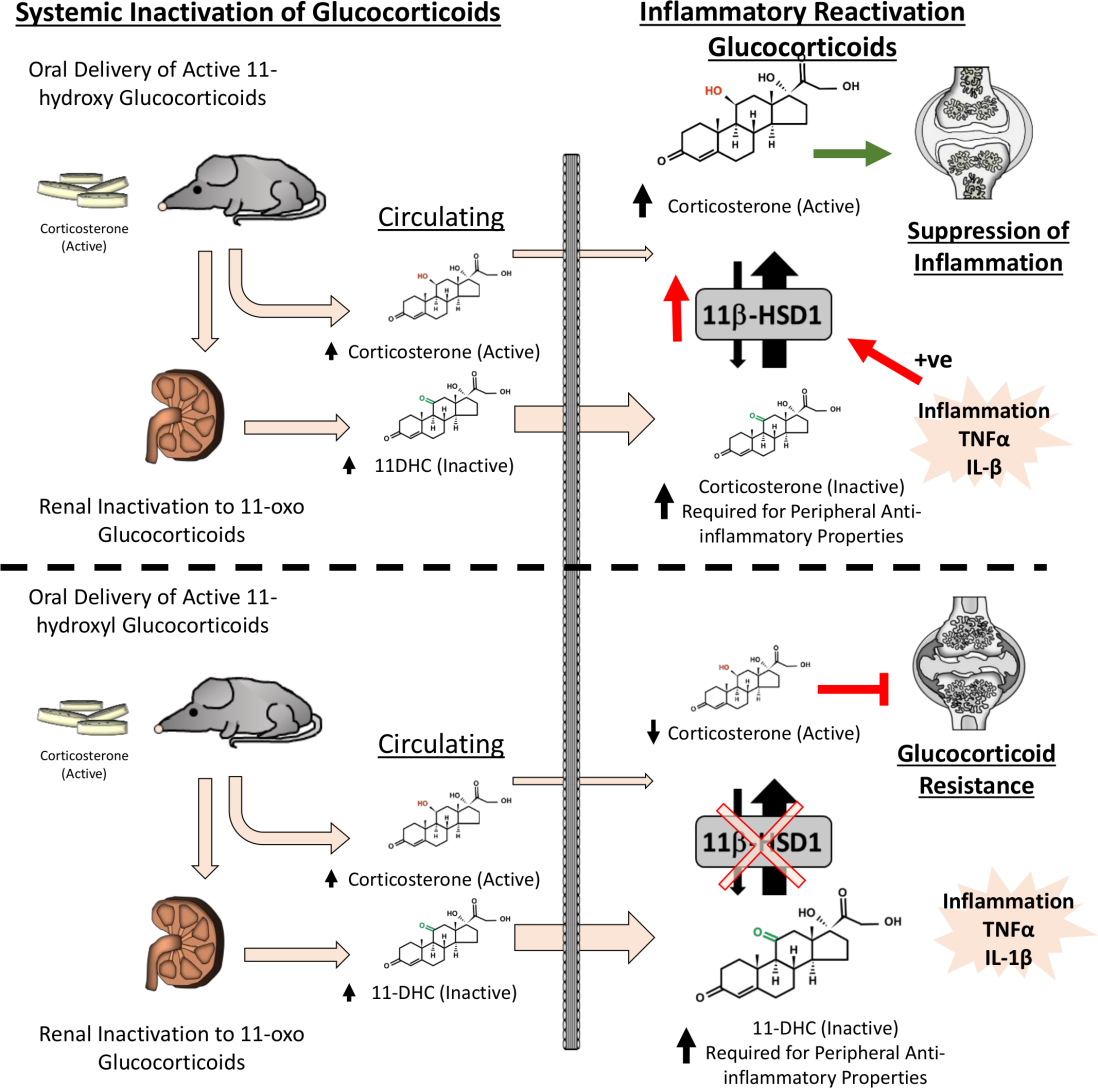
At therapeutic doses, the glucocorticoid corticosterone requires peripheral reactivation by 11β-hydroxysteroid dehydrogenase type 1 (11β-HSD1) at sites of inflammation, which is potently upregulated by pro-inflammatory factors such as tumour necrosis factor (TNF)-α and interleukin (IL)-1β, to enable their anti-inflammatory effects. The deletion of 11β-HSD1 (shown below the dashed line) prevents this critical step, resulting in severe GC resistance.

Despite the potent upregulation of 11β-HSD1 at sites of inflammation, its roles in mediating the effects of active therapeutic GCs have remained poorly understood.^19–22^ Here, studies by Schmidt *et al* and Hardy *et al* reported increasing levels of 11β-HSD1 within FLS and synovial macrophages that correlated with inflammation. Using murine models of polyarthritis, we have identified an entirely novel and, until now, unrecognised component of therapeutic GC action, whereby they require peripheral reactivation by 11β-HSD1 at sites of inflammation to deliver anti-inflammatory effects (figure 7). Here, the global transgenic deletion of 11β-HSD1 prevents this critical step, resulting in severe GC resistance in both TNF-tg and K/BxN models of polyarthritis.

The importance of endogenous GC metabolism by 11β-HSD1 in the pathophysiology of inflammatory polyarthritis are well established.^8 18 23^ We used oral corticosterone to suppress disease activity and joint inflammation in murine models of polyarthritis.^2 14 24^ We observed effective suppression at 100 µg/mL in the drinking water, where daily intake of corticosterone was 22.5 µg/g in the mice, and would be estimated to equate to administration of 40 mg hydrocortisone or 10 mg of prednisolone per day in an adult. Here, total active corticosterone and inactive 11-DHC, increased in TNF-tg and TNF‐tg^11βKO^ mice, with the reduced transcortin binding affinity of 11-DC predicted to further elevate its circulating free levels.^25^ In the models of polyarthritis, disease activity, synovitis and joint destruction were markedly suppressed in animals receiving corticosterone. However, we observed a profound GC resistance in 11β-HSD1 KO animals, despite equivalent serum exposure to corticosterone and 11-DHC. While these animals with deletion of 11β-HSD1 retained a capacity to respond to oral corticosterone, with evidence of a GC-mediated adrenal suppression and limited improvements in body weight and pain behaviour in the K/BxN model, these levels were insufficient to mediate anti-inflammatory actions within the joint. This indicates that the peripheral metabolism and activation of GCs such as corticosterone by 11β-HSD1 are required to mediate their anti-inflammatory properties. In this study, we used the GC corticosterone in our models as, within mice, it possesses equivalent action and metabolism as the steroid hydrocortisone in humans. Further research is now required to examine how synthetic GCs such as prednisolone and prednisone are metabolised by 11β-HSD1 at sites of inflammation in murine models and in human disease cohorts. This is of particular interest in human inflammatory disease, with Schmidt *et al* reporting shifts towards reduced synovial GC activation in synoviocytes in rheumatoid arthritis (RA) relative to osteoarthritis.^20^ This appeared to occur secondary to a shift in the 11β-HSD1/11β-HSD2 ratio, favouring steroid inactivation and was potentially attributed to a loss of sympathetic nerve fibre signalling to the RA joint. Analysis of synovial tissue shed light on the mechanism of GC resistance in the TNF‐tg^11βKO^ mouse. A key mechanism of action of therapeutic GC in the inflamed synovium is the suppression of leucocyte recruitment and reduction in pro-inflammatory cytokines and chemokines through suppression of pro-inflammatory pathways.^26–28^ In this feature of anti-inflammatory GC action, there remains ongoing debate in relation to the relative contributions of transactivation and transrepression as mechanism underlying the anti-inflammatory effects of GCs.^29^ In this study, we were unable to assess whether the anti-inflammatory properties of GC metabolism by 11β-HSD1 were predominantly mediated by transrepression or transactivation, which remains a prominent area of interest and the focus of prominent reviews in the field.^1^ In TNF‐tg^11βKO^ animals, expression of pro-inflammatory mediators persisted at sites of inflammation in response to corticosterone, with increased inflammatory activated M1-like polarisation, revealing a critical role for therapeutic GC metabolism by 11β-HSD1 in this process. These data suggest that in response to therapeutic GCs, 11β-HSD1 may mediates a shift from inflammatory activated M1-like macrophages to M2-like polarisation. However, the precise nature of these changes in this setting remains complex and is the subject, requiring more detailed characterisation, which in itself is the feature of a notable systematic review by Tardito *et al*.^30^ Of interest, we observed that several leucocyte population, including CD8 T cells and CD19 B cells within the synovium of 11β-HSD1 KO animals retained responsiveness to oral corticosterone and were suppressed to a similar degree as WT counterparts. These data indicate that certain leucocyte populations retain the capacity to respond to circulating levels of active corticosterone, present even in the absence of 11β-HSD1, suggesting they possess a lower GC receptor activation threshold that is independent of 11β-HSD1.

Given that FLS and macrophages highly express 11β-HSD1 in inflammatory environments, we examined whether targeted deletion of 11β-HSD1 within mesenchymal derived FLS and myeloid-derived macrophages could recapitulate global steroid resistance. Here, despite effective 11β-HSD1 deletion in FLS, TNF-tg^11βflx/tw2cre^ animals showed an entirely normal anti-inflammatory response to oral corticosterone, suggesting that GC reactivation within this subset alone was not critical to the anti-inflammatory properties of corticosterone. Similar findings were evident in myeloid-targeted TNF-tg^11βflx/LysMcre^. Here despite effective deletion of 11β-HSD1 within macrophages, TNF-tg^11βflx/LysMcre^ animals retained the capacity to respond to corticosterone. However, their response did appear to be muted, with disease activity scores being greater than TNF-tg counterparts, and with evidence of persistent joint destruction despite exposure to therapeutic GCs. The contribution of 11β-HSD1 within further leucocyte populations such as T cells to corticosterone resistance in the global KO deserve further scrutiny in this context but went beyond the scope of this study.

However, these data may suggest that the autocrine amplification of GCs by 11β-HSD1 within fibroblasts or macrophages alone is insufficient to mediate the anti-inflammatory actions of therapeutic GCs in vivo, as occurs with cell-targeted GC receptor KO studies.^31–33^ Instead, we explored whether paracrine signalling between macrophages and FLS might overcome cell-targeted deletion of 11β-HSD1 using in vitro models. These experiments revealed that metabolism of 11-DHC by 11β-HSD1 could mediate paracrine GC signalling between FLS and macrophages, compensating for cell-targeted deletion of 11β-HSD1 and reversing steroid resistance. Consequently, it may be that targeting any one cell population is insufficient to reproduce the phenotype observed with global 11β-HSD1 deletion. Ultimately, it is important to note that results observed in animal models are not always replicated in human disease, and these findings now require robust validation in patients with inflammatory disease.

In this study, we demonstrate a profound and previously unrecognised role for pre-receptor metabolism and activation of GCs by the enzyme 11β-HSD1 in mediating the anti-inflammatory therapeutic actions of oral GCs. Consequently, this study adds significant insight into our mechanistic understanding of therapeutic GC action. Here, a greater awareness of the how 11β-HSD1 targets the anti-inflammatory actions of therapeutic GCs at sites of inflammation may be able to inform the development of better-tolerated steroids that possess enhanced kinetics and activation efficiency by 11β-HSD1 to improve targeting and dosing efficacy, as well as informing ongoing studies examining the application of therapeutic 11β-HSD1 inhibitors.
