# Supplementary material for: Local steroid activation is a critical mediator of the anti-inflammatory actions of therapeutic glucocorticoids
Source: Ann Rheum Dis. 2020 Nov 8;80(2):250–60. doi: 10.1136/annrheumdis-2020-218493 (PMC7815637; doi:10.1136/annrheumdis-2020-218493)
Supplement: Supplementary data [file annrheumdis-2020-218493supp001.pdf]

| Target | Label | Dilution | Manufacturer | Reference | Concentration |
|---|---|---|---|---|---|
| anti-CD45 | APC-CY7 | 1:400 | eBioscience | 47-0451-82 | 0.2mg/ml |
| anti- CD11b | PerCP CY5.5 | 1:200 | BioLegend | 45-0112-82 | 0.2mg/ml |
| anti-CD11c | FITC | 1:100 | eBioscience | 11-0114-82 | 0.5mg/ml |
| anti-SiglecF | eFlour660 | 1:100 | eBioscience | 50-1702-80 | 0.2mg/ml |
| anti-CD64 | PE-Cy7 | 1:100 | BioLegend | 139314 | 0.2mg/ml |
| anti-Ly6g | PE Dazzle | 1:800 | BioLegend | 127648 | 0.2mg/ml |
| anti-Ly6c | BV510 | 1:600 | BioLegend | 128033 | 0.2mg/ml |
| anti-MHC Class II | BV711 | 1:800 | Biolegend | 107643 | 0.2mg/ml |
| anti-F4/80 | PE labelled | 1:400 | BioLegend | 123110 | 0.2mg/ml |
| anti-CD45 | APC-Cy7 | 1:400 | eBioscience | 47-0451-82 | 0.2mg/ml |
| anti-CD3 | PE-Cy7 | 1:400 | eBioscience | 25-0031-82 | 0.2mg/ml |
| anti-CD4 | Pacific blue | 1:600 | eBioscience | 116008 | 0.5mg/ml |
| anti-CD19 | PE labelled | 1:400 | eBioscience | 12-0193-82 | 0.2mg/ml |
| anti-CD8 | Texas red | 1:800 | eBioscience | MCD0817 | 0.2mg/ml |

Supplementary table 1: Antibodies for FACS analysis of synovial leukocyte populations. The following gating strategy was used for myeloid cells: Live cells were gated on CD45+CD11b+ cells. Neutrophils were identified as CD45+ CD11b + SiglecF- Ly6ghi, macrophages were CD45+ CD11b+ SiglecF- Ly6g- CD64+ F4/80+ and M1 macrophages were CD45+ CD11b+ SiglecF- Ly6g- CD64+ F4/80+ MHC Class II+. T cells were identified as live CD45+CD3+. CD3+ cells were then stratified as CD4+ or CD8+ T cells. B cells were identified as CD45+ CD3- and CD19+.
