# Supplementary material for: Local steroid activation is a critical mediator of the anti-inflammatory actions of therapeutic glucocorticoids
Source: Ann Rheum Dis. 2020 Nov 8;80(2):250–60. doi: 10.1136/annrheumdis-2020-218493 (PMC7815637; doi:10.1136/annrheumdis-2020-218493)
Supplement: Supplementary data [file annrheumdis-2020-218493supp002.pdf]

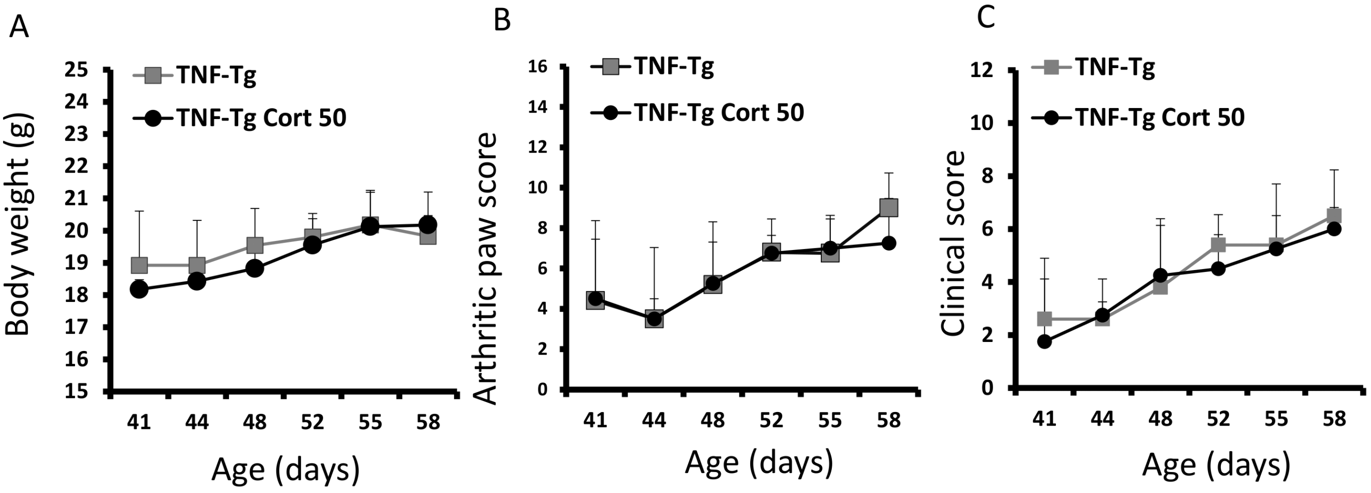

***Supplementary Figure 1**: (A), Body weights, (B) Total clinical scores (AU), (C) arthritic paw scores (AU) of WT, TNF-tg animals receiving either vehicle or corticosterone (50 µg/ml) in the drinking water for 3 weeks. Values are expressed as mean ± standard error, n=3 per group. Statistical significance was determined using two-way ANOVA with Tukey post hoc analysis. *P < 0.05, **P < 0.005, ***P < 0.001.*
