# Supplementary figures and images for: Local steroid activation is a critical mediator of the anti-inflammatory actions of therapeutic glucocorticoids
Source: Ann Rheum Dis. 2020 Nov 8;80(2):250–60. doi: 10.1136/annrheumdis-2020-218493 (PMC7815637; doi:10.1136/annrheumdis-2020-218493)

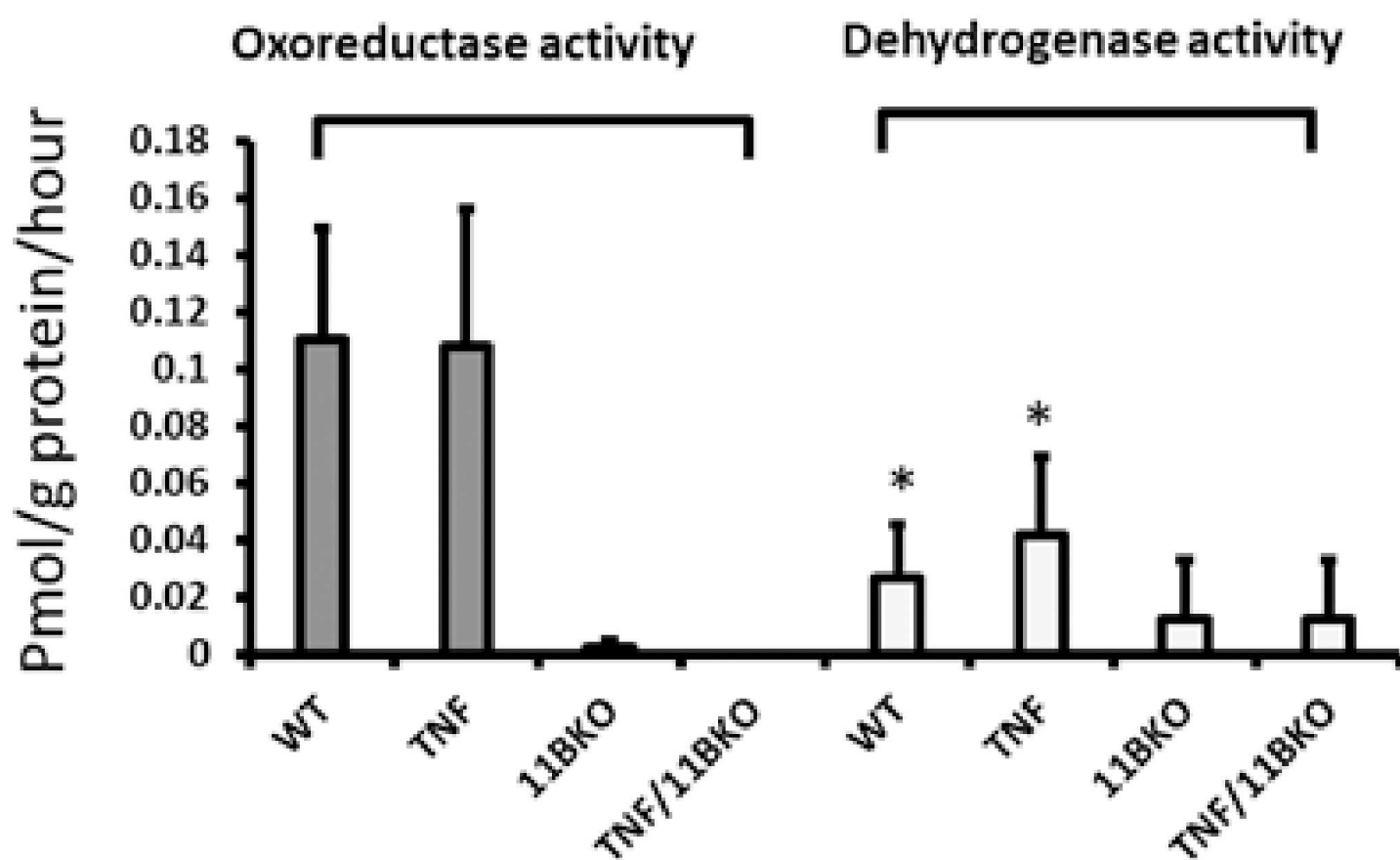

Supplement: Supplementary data [file annrheumdis-2020-218493supp003.pdf]
